# Supplementary material for: CRISPR/Cas9-Based Knock-Out of the PMR4 Gene Reduces Susceptibility to Late Blight in Two Tomato Cultivars
Source: Int J Mol Sci. 2022 Nov 22;23(23):14542. doi: 10.3390/ijms232314542 (PMC9735651; doi:10.3390/ijms232314542)
Supplement: Supplementary file 1 [file ijms-23-14542-s001.zip › ijms-2000467-supplementary.pdf]

Supplementary Table S1 - Primers used in PCR and RT-qPCR

Objective	Primer name	Region screened	Forward primer (5' - 3')	Reverse primer (5' - 3')	Amplicon size (bp)
Cas9 amplification	Cas9F;Cas9R	Cas9	CTATCCTCAGGCGGCAAGAG	AGTCATCCACGCGAATCTGG	137
Actin amplification	ActinF; ActinR	Actin	TCCGCGACATGAAGGAAAAGC	GCAACGGAACCTCTCAGCAC	146
Big deletion detecting	sg8F; sg7R	sgRNA8 + sgRNA1 + sgRNA7	GCGAATGCGTAGAGAAGGAA	CCCCACTAAGTGCCAGGTAA	1246
Big deletion detecting	sg6F; sg7R	sgRNA6 + sgRNA8 + sgRNA1 + sgRNA7	GCTTTTCTGAATCGGATCGTA	CCCCACTAAGTGCCAGGTAA	3564
TIDE	tide_sg6F; tide_sg6R	sgRNA6	GCTTTTCTGAATCGGATCGTA	ATTCCTGCATCAAGTAACGAC	440
TIDE	tide_sg8F; tide_sg1R	sgRNA8 + sgRNA1	AAATTTCTTGCAGCGAATGCGTAG	GGCCTTTAAACAACATACTCAC	470
TIDE	tide_sg7F; sg7R	sgRNA7	GTTCCGTGTCATCCCTTGCT	CCCCACTAAGTGCCAGGTAA	492

Supplementary Table S2 -Assembly statistics of selected edited and control plants of San Marzano genotype

	SM6	SM13	SM17	SM19	CTRL
**N° scaffold**	390,956	408,513	403,795	391,614	415,830
**assembled genome (Mb)**	830.9	833.7	832.8	831.5	843.1
**Mean scaffold size (bp)**	2,125	2,041	2,062	2,123	2,027
**N50 scaffold length (bp)**	28,863	28,271	27,917	31,529	30,452
**L50 scaffold count**	7,286	7,416	7,556	6,621	6,913
**Read coverage over Solyc10g009390.3 (single copy gene)**	45.1	33.3	32.8	36.6	36.9
**Read coverage over Cas9 gene**	45.5	30.2	36.1	36.5	-
**Inferred N° copies *Cas9* (hemyzigous)**	2.0	1.8	2.2	2.0	-
**N° copies *Cas9* (qPCR)**	2.2	2.0	1.8	1.8	-

Supplementary Table S3 - Details of the indels produced by the 4 sgRNAs in each edited line

Mutant	Alleles in sgRNA7 region (%)		R^2
	**-7**	**0**	
**SM4**	95	-	0,98
**SM5**	91,9	-	0,98
**SM6**	97,5	-	0,99
**SM13**	96	-	0,99
**SM17**	97,8	-	0,98
**SM19**	97,9	-	0,99
**SM22**	96,6	-	0,98
**OX2**	95,6	-	0,99
**OX3**	97,7	-	0,99
**OX4**	98,5	-	0,99
**OX9**	98,6	-	0,99
**OX11**	98	-	0,99

Mutant	Alleles in sgRNA8 region (%)				R^2
	**-7**	**-2**	**0**	**1**	
**SM4**	54.2	25.5	-	34.6	0,9
**SM6**	46,7	48,4	-	-	0,95
**SM7**	46,1	48,4	-	-	0,94
**SM8**	43,6	51,6	-	-	0,96
**SM9**	44,8	50,6	-	-	0,95
**SM12**	45	48,3	-	-	0,93
**SM13**	43,4	45,8	-	-	0,9
**SM14**	41,9	50,9	-	-	0,93
**SM16**	-	-	-	96,1	0,96
**SM17**	43,2	50,1	-	-	0,94
**SM18**	44,5	48	-	-	0,93
**SM19**	45,1	47,7	-	-	0,93
**SM22**	45,4	48,4	-	-	0,94
**SM24**	44,9	48,7	-	-	0,94
**SM25**	45,1	49,1	-	-	0,93
**SM26**	44,5	49,4	-	-	0,94
**OX2**	-	96.5	-	-	0,99
**OX3**	-	-	-	95	0,98
**OX4**	-	96,2	-	-	0,99
**OX9**	-	69.5	-	26,4	0,96
**OX11**	-	91,3	-	-	0,95

Mutant	Alleles in sgRNA6 region (%)										R^2
	-18	-8	-7	-5	-4	-3	-2	-1	0	1	
**SM1**	-	-	-	-	-	-	-	-	-	-	0,01
**SM2**	-	-	-	-	-	1,6	-	-	96,8	-	0,99
**SM3**	-	-	-	-	-	7.2	0.8	-	90.5	-	0,99
**SM4**	-	-	-	-	-	2.9	-	-	57.6	34.6	0,96
**SM5**	-	-	-	-	-	-	-	-	71.7	24.2	0,96
**SM6**	-	-	11.4	12	-	-	16.8	-	54.8	-	0,95
**SM7**	-	-	-	-	8.6	-	-	-	85.6	1.4	0,99
**SM8**	-	-	-	-	-	-	-	10.9	61.8	21.1	0,96
**SM13**	27,5	-	-	12,2	-	8,1	-	-	-	38,8	0,93
**SM17**	-	-	31,7	-	-	-	-	-	28,2	31,7	0,93
**SM19**	-	-	-	-	-	-	-	-	97	-	0,98
**SM22**	-	-	-	-	4	6,3	-	5,7	28,6	51,4	0,96
**OX1**	-	-	-	-	15	-	-	-	41.8	39.7	0,97
**OX2**	-	-	-	-	8.4	-	-	-	80.8	9.1	0,99
**OX3**	-	-	-	-	-	-	26.8	-	69.6	-	0,96
**OX4**	-	6.6	-	-	-	-	-	-	84.7	6.6	0,99
**OX9**	-	-	-	-	-	6,2	-	-	84,4	5,9	0,99
**OX11**	-	-	-	-	-	-	-	48.5	46,1	-	0,95

Mutant	Alleles in sgRNA1 region (%)										R
	-10	-8	-7	-6	-5	-3	-2	-1	0	1	
**SM6**									92,2		0,96
**SM7**							52,6		35,8		0,94
**SM8**	8,4				10,6			14,3	59,3		0,93
**SM9**	-	2	11.4	-	-	-	7	6.9	9.3	3.4	0,45
**SM13**				45,7					43,9		0,92
**SM17**							54,7	15	18,3	3,2	0,94
**SM18**		45,3		17,8					8,4	12,2	0,88
**SM19**		27,6							60		0,91
**SM24**				17,6					57,4	9,8	0,92
**OX3**		31,8							-		0,57
**OX11**		10,3	-	-		16,7			60	-	0,93

Supplementary Table S4 - Detached-leaves assay with P. infestans performed on 26 PMR4 San Marzano mutants, 9 Oxheart mutants and control plants. Two independent experiments were separately conducted. LAD% values here reported were normalized using LAD% derived from the controls. Statistical differences among mutant/control were analyzed with a two-tailed t test (*, p < 0.05).

	1° experiment				2° experiment			
**Genotype**	**mean LAD%**	**st. deviation**	**p-value**	**sig.**	**mean LAD%**	**st. deviation**	**p-value**	**sig.**
**SM1**	0.57	0.14	0.021	*	1.13	0.47	0.687	
**SM2**	0.57	0.14	0.021	*	-	-	-	
**SM3**	0.28	0.14	0.004	*	1.43	0.47	0.254	
**SM4**	0.14	0.07	0.003	*	0.20	0.09	0.000	*
**SM5**	1.20	0.20	0.059		-	-	-	
**SM6**	0.60	0.20	0.049	*	0.20	0.09	0.000	*
**SM7**	0.80	0.04	0.192		0.82	0.18	0.207	
**SM8**	1.00	0.20	1.000		0.82	0.18	0.207	
**SM9**	0.66	0.46	0.343		0.19	0.19	0.011	*
**SM10**	0.60	0.53	0.325		0.56	0.50	0.267	
**SM11**	0.26	0.12	0.001	*	0.75	0.00	0.225	
**SM12**	1.26	0.95	0.674		0.06	0.11	0.009	*
**SM13**	0.26	0.00	0.007	*	0.29	0.25	0.019	*
**SM14**	0.44	0.31	0.097		0.69	0.30	0.224	
**SM15**	0.44	0.31	0.097		1.29	0.74	0.577	
**SM16**	0.71	0.15	0.054		1.43	0.49	0.279	
**SM17**	0.34	0.06	0.002	*	0.35	0.00	0.031	*
**SM18**	0.44	0.11	0.003	*	1.00	0.27	0.185	
**SM19**	0.19	0.00	0.003	*	0.06	0.07	0.003	*
**SM20**	-	-	-		0.32	0.29	0.059	
**SM22**	0.28	0.00	0.007	*	1.77	0.74	0.221	
**SM23**	1.04	0.72	0.921		0.62	0.13	0.036	*
**SM24**	0.47	0.16	0.014	*	0.92	0.40	0.787	
**SM25**	1.04	0.16	0.725		0.69	0.00	0.087	
**SM26**	1.42	0.29	0.103		1.70	0.62	0.187	
**CTRL-SM**	1.00	0.13	-		1.00	0.12	-	
**OX1**	0.30	0.00	0.019	*	0.67	0.13	0.095	
**OX2**	0.60	0.30	0,139		0.67	0.13	0.096	
**OX3**	0.20	0.17	0.005	*	0.53	0.27	0.072	
**OX4**	0.00	0.00	0.001	*	0.27	0.18	0.009	*
**OX5**	0.80	0.46	0.530		0.76	0.20	0.212	
**OX6**	0.78	0.21	0.232		0.56	0.10	0.041	*
**OX7**	0.85	0.09	0.272		0.98	0.15	0.886	
**OX8**	0.75	0.15	0.132		0.71	0.15	0.118	
**OX9**	0.70	0.17	0.101		0.80	0.27	0.357	
**CTRL-OX**	1.00	0.17	-		1.00	0.20	-	

Supplementary Table S5 - Sequencing stats in the 4 edited mutants and in the WT San Marzano genotypes.

Sample	Raw reads	Raw data (Gb)	Effective (%)	Error (%)	Q20 (%)	Q30 (%)	GC (%)	Coverage
**SM-6**	271,306,910	40.70	99.33	0.03	97.34	93.06	36.39	52.01
**SM-13**	198,386,556	29.80	99.32	0.03	97.50	93.14	37.22	38.08
**SM-17**	227,401,788	34.10	99.35	0.03	97.52	91.15	37.14	43.58
**SM-19**	221,574,476	33.20	99.29	0.03	97.22	92.59	36.46	42.43
**SM-CTRL**	223,484,784	33.50	99.29	0.03	97.19	92.55	36.53	42.81
**SM-WT-1**	259,976,742	39.00	99.01	0.03	97.17	92.01	36.26	49.84
**SM-WT-2**	275,668,832	41.40	99.14	0.03	96.57	91.01	36.16	52.91

Supplementary Table S6 - Off-target regions and sgRNA-like and SNP/indels found in the edited mutants and in the San Marzano genotype (CTRL).

Off-target genomic coordinates	strand	off-target sequence	PMR4 sgRNA	Type of mismatches	PAM	N° of mismatches	in CDS	N° SNP/indel				
								SM6	SM13	SM17	SM19	CTRL
SL4.0ch02:40968952-40968975	-	GCtAAGGT_TGCtAGTGGtAA-TGGT	7	A21	A	3	yes	0	0	0	0	0
SL4.0ch09:32079813-32079836	-	GtCAAtGT_TGCCAaTGaCAA-TGGT	7	A22	A	4	-	0	0	0	0	0
SL4.0ch10:29190858-29190881	+	GtCAAGcT_TGaCAGTGGtAA-TGGT	7	A22	A	4	-	0	0	0	0	0
SL4.0ch12:27868456-27868479	+	GaCAAtGT_TGtCAGTtGCAA-TGGG	7	A22	A	4	-	0	0	0	0	0
SL4.0ch02:22578974-22578997	+	TTAcAGgA_GTCCCAaACTCc-GGGA	1	A22	A	4	yes	0	0	0	0	0
SL4.0ch04:33536140-33536163	+	TTgAAGCt_cTCCCATAgTCG-AGGC	1	A22	A	4	-	0	0	0	0	0
SL4.0ch05:27723956-27723979	-	TaAAAGgA_GTCCCATgCTCa-TGGA	1	A22	A	4	yes	0	0	0	0	0
SL4.0ch08:47322517-47322540	-	TTgAAGCg_GTgCCATACTCc-TGGT	1	A22	A	4	-	0	0	0	0	0
SL4.0ch09:3305746-3305769	-	TTAAAaCA_GTCgCATtCTCG-GGGA	1	A21	A	3	-	0	0	0	0	0
SL4.0ch01:85268760-85268783	-	GGATtTgA_GAGAAGtAcCAG-TGGG	8	A22	A	4	-	0	0	0	0	0
SL4.0ch02:15897577-15897600	+	GGtTATtA_GAGAAGGATCAt-GGGA	8	A12	A	3	-	0	0	0	0	0
SL4.0ch03:19724933-19724956	+	GGAaATaA_GAGAgGGATCAt-AGGG	8	A22	A	4	-	0	0	0	0	0
SL4.0ch05:15677408-15677431	-	GGtTgTCA_aAGAAGaATCAG-TGGA	8	A22	A	4	-	0	0	0	0	0
SL4.0ch07:29758891-29758914	-	GGATAaCg_GAGAAGGAgaAG-AGGA	8	A22	A	4	-	0	0	0	0	0
SL4.0ch10:49269193-49269216	+	GcAgATCA_cAGAAGtATCAG-AGGT	8	A22	A	4	-	0	0	0	0	0
SL4.0ch03:24947651-24947674	+	aTAaTGCC_CCACACTgTGta-AGGT	6	A32	A	5	-	0	0	0	0	0
SL4.0ch04:16536110-16536133	+	tTAtTGCC_CCAtACTCTcCa-TGGA	6	A32	A	5	-	0	0	0	0	0
SL4.0ch05:47540410-47540433	-	GTAtTtCC_tCACtCTCTtCG-AGGA	6	A32	A	5	-	0	0	0	0	0
SL4.0ch05:58288753-58288776	-	GaACTGtC_CCACACctTGCa-GGGA	6	A32	A	5	-	0	0	0	0	0
SL4.0ch05:64845772-64845795	-	GTgCTGtC_CCACttTCTGgG-AGGC	6	A32	A	5	yes	0	0	0	0	0
SL4.0ch07:10476402-10476425	+	GTACTGga_CCACtgTCTGCa-TGGA	6	A32	A	5	-	0	0	0	0	0
SL4.0ch08:58075701-58075724	+	GTACTGaa_CCtgAtTCTGCG-TGGT	6	A32	A	5	yes	0	0	0	0	0
SL4.0ch09:46513077-46513100	+	GTcgTGCC_CCcCAtTgTGCG-AGGA	6	A32	A	5	-	0	0	0	0	0
SL4.0ch09:64797758-64797781	-	tTAtTGCC_tCACACatTGCG-AGGC	6	A32	A	5	yes	0	0	0	0	0
SL4.0ch12:60823071-60823094	+	GTACTGat_CtAtACTCTcCG-CGGA	6	A32	A	5	yes	0	0	0	0	0

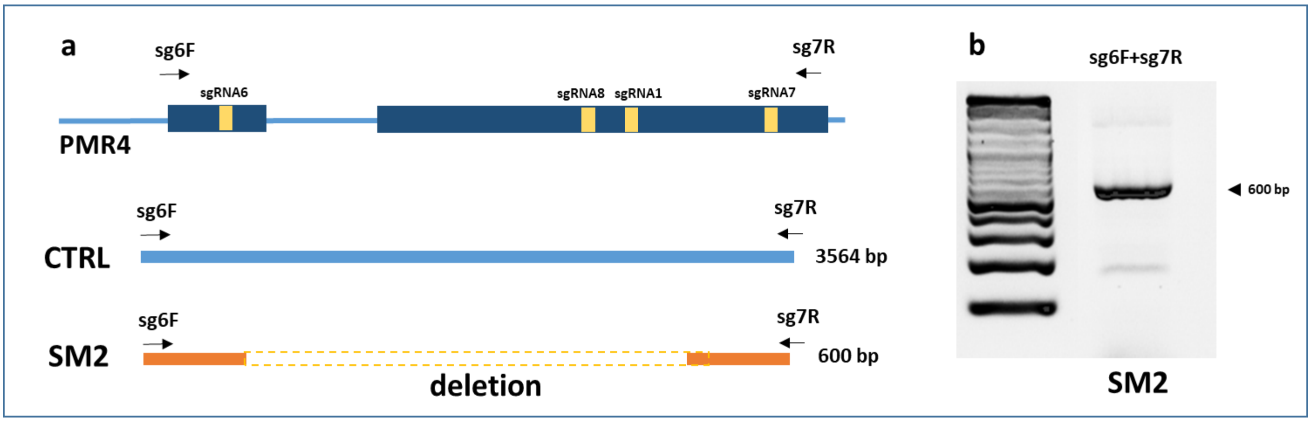

Supplementary Figure S1 - Large deletion observed in the *PMR4* gene in the SM2 mutant. (A) Scheme of the locus structure in SM2 and control plant. (B) PCR amplification of the mutant with primers sg6F and sg7R.

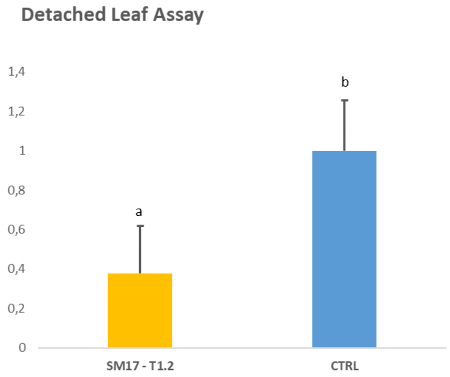

Supplementary Figure S2 - (a) Detached-leaves assay with *Phytophthora infestans* performed on T1 *PMR4* San Marzano mutant (SM17-T1.2) and a wild type plant as a control group at 8 dpi. (b) In the histogram, normalized LAD% values are reported for each genotype. The y-axis shows the mean ratio of the score of the mutant/control group; bars represent standard deviation (sd). Statistical differences among mutant/control were analyzed with a two-tailed t test ($P < 0.05$).

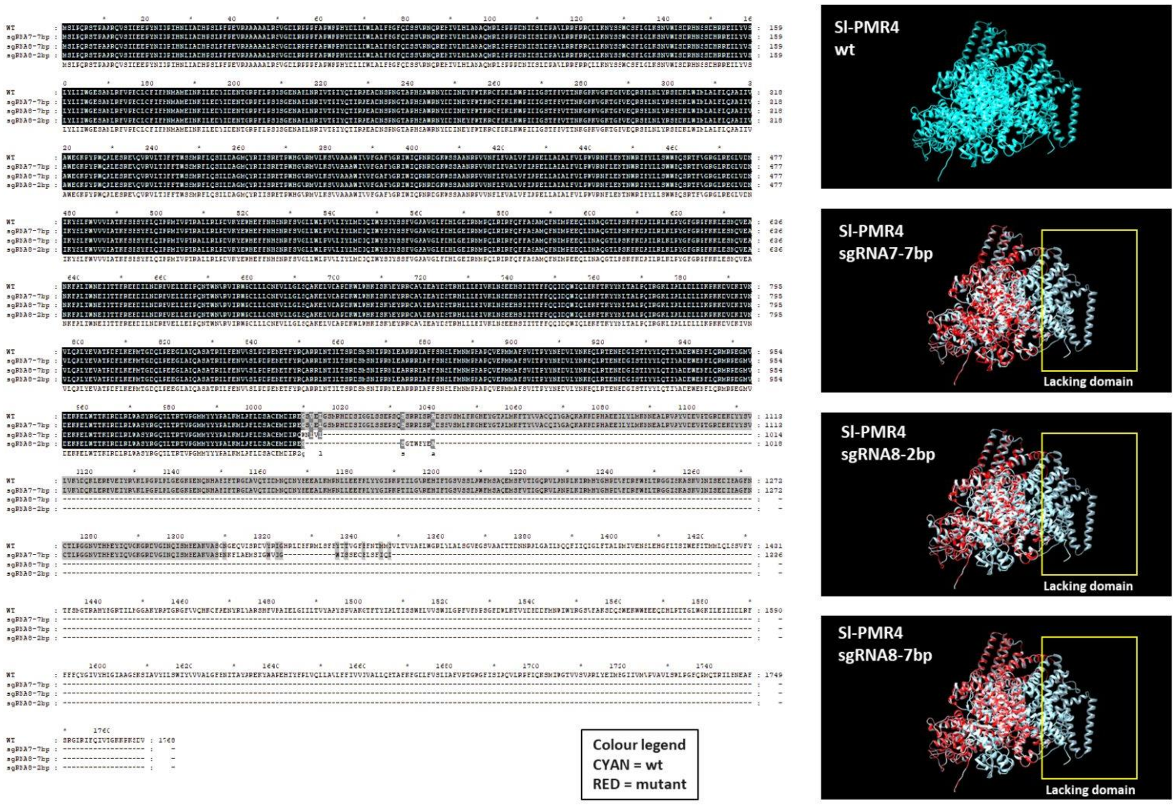

Supplementary Figure S3 - Multi-alignment of PMR4 proteins carrying the indels observed in the edited mutants (SM6, 13, 17, 17-T1.2, 19), highlighting truncated versions of the wild-type protein. Pairwise juxtaposition of the 3D-protein models (WT and mutated PMR4) through the UCSF Chimera tool.

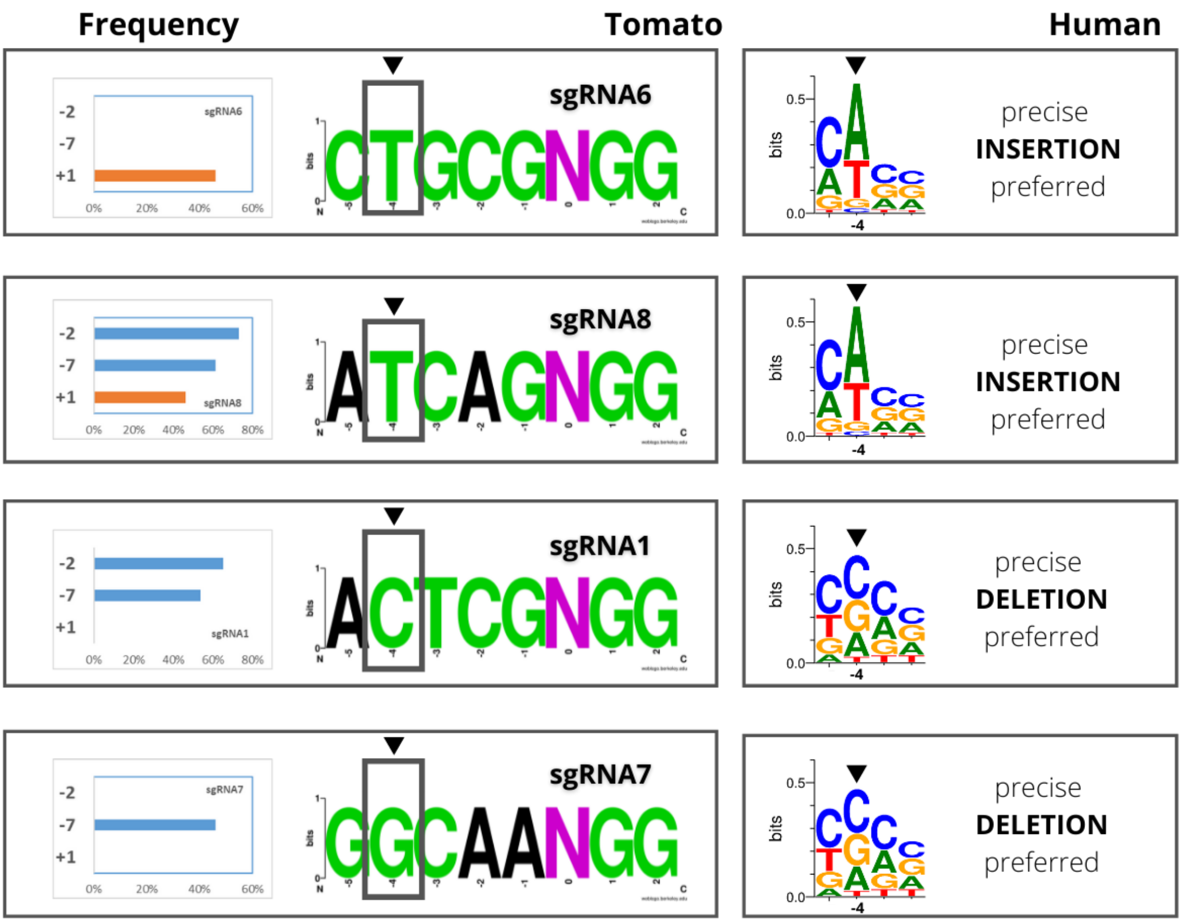

Supplementary Figure S4 - Sequence logos for the precision core of the four sgRNAs used in this study. Left: Frequency of the most common indels (insertions or deletions) within the whole set of edited plants (SM and OX); details of the flanking PAM sequence with the −4 nucleotide position highlighted in a square box. Right: statistics reported in human for RNA-guided Cas9 nuclease (RGN) preferred indels (Chakrabarti et al. 2019).

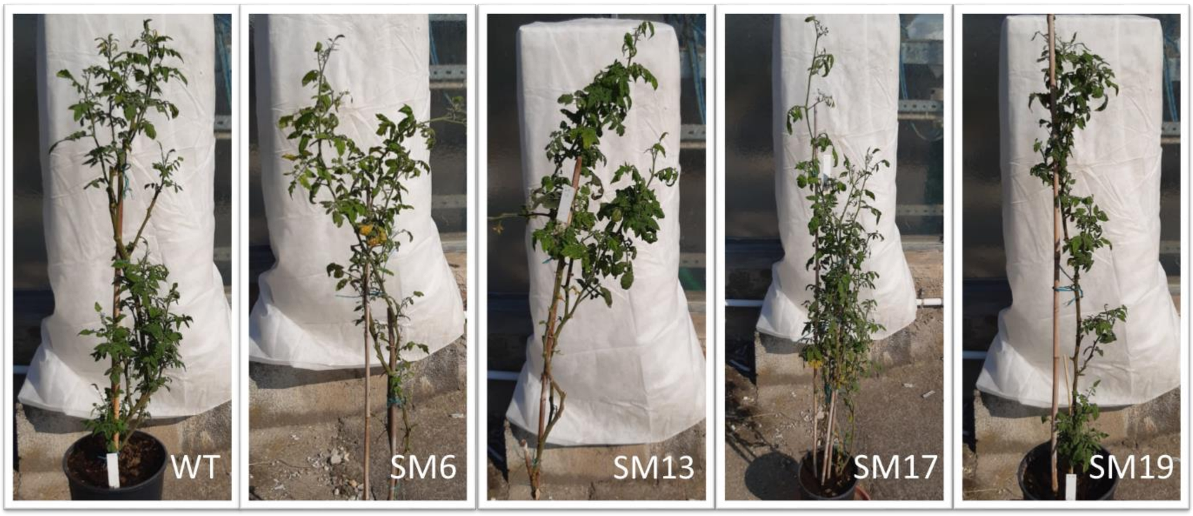

Supplementary Figure S5 - *pmr4* San Marzano mutants (SM6, 13, 17, and 19) and the wt plant in cultivation (9 months old).
